# Supplementary material for: The Impact of Non-Adherence to Antihypertensive Drug Therapy
Source: Healthcare (Basel). 2023 Nov 18;11(22):2979. doi: 10.3390/healthcare11222979 (PMC10671374; doi:10.3390/healthcare11222979)
Supplement: Supplementary file 1 [file healthcare-11-02979-s001.zip › healthcare-2654936-supplementary.pdf]

Supplemental Table S1. Summary Statistics:

	Number of Counties	Mean	SD	Minimum	Maximum
**BP Nonadherence** (%)	3196	26.59	5.34	15.90	56.20
**Diuretic Nonadherence** (%)	3213	28.77	4.68	16.40	51.40
**RAAS Nonadherence** (%)	3213	20.26	3.95	12.20	49.60
**CVA Death All** (%)	3212	77.76	16.78	17.50	208.90
**CVA Hospitalizations All** (%)	3209	10.64	2.68	1.60	23.00
**Total CVD Deaths All** (%)	3217	467.27	101.72	147.70	1132.80
**Total CVD Hospitalizations All** (%)	3210	61.30	17.51	17.90	135.10
**HTN Deaths All** (%)	3217	233.48	97.90	32.20	933.80
**HTN Hospitalizations All** (%)	3082	2.61	1.49	0.20	16.20
**Insured** (%)	3134	12.02	5.07	2.10	37.40
**Medicaid Eligible** (%)	2917	23.05	8.42	0.60	62.00
**No High School** (%)	3213	14.95	7.04	1.60	53.70
**No College** (%)	3213	79.59	8.95	21.20	98.10
**Female HOH** (%)	3213	17.30	6.91	0.00	52.20
**Food stamp** (%)	3134	14.98	7.65	0.20	56.40
**Median Home Value** (%)	3211	132.97	77.14	32.30	902.50
**Median Household Income** (%)	3134	48.60	12.35	22.90	125.90
**Income Inequality (Gini Coefficient)**	3213	0.44	0.04	0.33	0.62
**Poverty** (%)	3134	16.27	6.44	3.40	47.40
**Unemployment** (%)	3212	4.53	2.11	1.20	21.40
